# Pain during and after coronavirus disease 2019: Chinese perspectives

**DOI:** 10.1097/PR9.0000000000000931

**Published:** 2021-05-10

**Authors:** Feng Jiang, Wan-Li Yang, Jia-Wei Wang, Zhen Zhu, Ceng Luo, Lars Arendt-Nielsen, Xue-Jun Song

**Affiliations:** a Translational Institute for Cancer Pain, Xinhua Hospital Affiliated to Shanghai Jiao Tong University School of Medicine, Chongming Branch, Shanghai, China; b School of Life Sciences, Shanghai University, Shanghai, China; c Department of Neurobiology, School of Basic Medicine, Fourth Military Medical University, Xi'an, China; d Center for Neuroplasticity and Pain (CNAP), SMI, Department of Health Science and Technology, School of Medicine, Aalborg University, Aalborg, Denmark; e SUSTech Center for Pain Medicine, School of Medicine, Southern University of Science and Technology, Shenzhen, Guangdong, China

**Keywords:** Global pandemic, COVID-19, Pain management, SARS-CoV-2

## Abstract

The coronavirus disease 2019 (COVID-19) global pandemic poses a major threat to human health and health care systems. Urgent prevention and control measures have obstructed patients' access to pain treatment, and many patients with pain have been unable to receive adequate and timely medical services. Many patients with COVID-19 report painful symptoms including headache, muscle pain, and chest pain during the initial phase of the disease. Persistent pain sequela in patients with COVID-19 has a physical or mental impact and may also affect the immune, endocrine, and other systems. However, the management and treatment of neurological symptoms such as pain are often neglected for patients hospitalized with COVID-19. Based on the China's early experience in the management of COVID-19 symptoms, the possible negative effects of pre-existing chronic pain in patients with COVID-19 and the challenges of COVID-19 prevention and control bring to the diagnosis and treatment of chronic pain are discussed. This review calls to attention the need to optimize pain management during and after COVID-19.

## Abstract 2

Pain during and after COVID-19 is common. In China, infection spread was swiftly brought under control, and pain management was incorporated into widely accessible treatment protocols combining traditional Chinese medicine and modern medicine.

## 1. Introduction

The coronavirus disease 2019 (COVID-19) pandemic has dramatically changed the way people live around the world. In particular, it has had a profound impact on normal medical activities and required repositioning health care personnel to front line services. To cope with the risk of hospital-acquired infection, medical institutions have had to multiply health care costs and resource use.^5^ However, patients with pain may not get better care despite the increased health care spending. Importantly, to reallocate resources to the emergency department, many nonemergency departments at medical institutions have been completely or partly closed, including many pain management departments.^16^ This review, based on the Chinese experience, attempts to uncover the increased risk of pain that patients with COVID-19 may face and outline that pain may not only be a symptom of COVID-19 but also one of the risk factors that influence the improvement of COVID-19 and other patients. This review will highlight and discuss China's early experience in the management of COVID-19 symptoms, including pain, and apply this in a global context.

### 1.1. COVID-19 infection increases the risk of pain

Although pulmonary infection is the major symptom of SARS-CoV-2 infection, more than a third of the patients with COVID-19 had different neurological symptoms which may involve the central nervous system (dizziness, headache, impaired consciousness, ataxia, and epilepsy) and the peripheral nervous system (sensory system impairments and neuralgia) and skeletal muscle damage/pain or chest pain.^41^ In some patients, neurological symptoms may be the first or only manifestation of COVID-19.^2^ In addition, patients with more severe SARS-CoV-2 infection are more likely to show neurological symptoms.^41^ Although severe pain is rare, different forms of pain are important clinical manifestations. In a large study based on 1,099 patients (926 nonsevere and 173 severe) collected in 552 Chinese hospitals, 150 (13.6%) had headache and 164 (14.9%) had muscle or/and joint 30 pain and 153 (13.9%) with sore throat.^24^ Because of the differences in study objectives, a very wide range of pain prevalence was reported.

### 1.2. Headache

Headache is one of the most frequent forms of pain during SARS-CoV-2 infection. The exact incidence of headaches caused by the virus needs further study, while most studies suggest headache prevalence ranged between 6% and 21%.^15,58^ Whether the headache is caused by the virus invading the nervous system directly or indirectly remains uncertain. An autopsy report showed damage to the central nervous system (CNS) as evidenced by brain edema and partial neuronal degeneration.^63^ The presence of SARS-CoV-2 in the cerebrospinal fluid was confirmed by genome sequencing, indicating that the virus may penetrate and cause damage to the CNS.^4^ Existing knowledge about coronaviruses suggests that the coronavirus family has a certain tropism for the CNS.^8^ Neuronal pathways allow the virus to enter the CNS.^1^ The virus can infect sensory and motor nerve endings to migrate, then achieve retrograde or anterograde transport by movement protein, dynein protein, and dynamic neurons to invade nerve tissues and cause infection of immunocompetent microglia, astrocytes, and macrophages in the CNS.^3,56,57^

### 1.3. Muscle pain

Muscle pain is another common symptom in patients with COVID-19. When comparing the existing studies, the incidence of muscle pain ranged between 7% and 32%.^24,36,66^ Since the target receptor of SARS-CoV-2, ACE2, is present in almost all tissues, including muscle tissues, the virus can attack skeletal muscle cells directly, causing skeletal muscle weakness, fatigue, pain, and injury.^20,27,43,53^ Rhabdomyolysis has been found in patients with severe SARS-CoV-2 infection.^13,19,29,58^ Apart from the direct cause, the inflammatory response induced by SARS-CoV-2 infection may be another important cause of muscle pain.^59^ Muscle injury caused by COVID-19 is characterized by a significant increase in the levels of creatine kinase and lactate dehydrogenase, and its pathophysiology is primarily believed to be caused by excessive inflammation.^33^

### 1.4. Chest pain

Chest pain, while relatively rare, generally occurred in patients with more severe symptoms especially when lesions are close to the pleura with abundant nerves.^7^ A descriptive study of the epidemiology and clinical characteristics of 99 cases of new coronavirus pneumonia in Wuhan in 2019 showed that when admitted to the hospital, most patients had fever or cough, and some patients had chest pain (2%).^11^ Compared with the patients with mild symptoms, the incidence of chest pain and dyspnea is higher in critical patients. In the severe patients, chest pain may be caused by the inflammatory reaction of the pleura. Their alveolar tissue is severely damaged, resulting in dyspnea.

### 1.5. Other kinds of pain

Other types of pain are also found during the process of clinical diagnosis. Researchers from Anglia Ruskin University found that among 83 interviewees, there was a significant increase in eye pain in the confirmed patients; 16% reported eye pain as one of their symptoms.^34^ Sore throat was reported in 5% to 17.4% of patients with COVID-19 in China.^11,21,60^ Of 76 patients with acute abdominal pain, 9 patients (11.8%) were eventually diagnosed with COVID-19.^54^

### 1.6. Pain sequela after COVID-19 in patients with or without chronic pain

Those patients with COVID-19 who experience intense pain and distress during their stay in the intensive care unit (ICU) seem to be at a higher risk of developing chronic pain after discharge.^32,39^ Unfortunately, in the ICU, pain is often overlooked as being low priority and thus poorly managed, even in a well-staffed and skilled environment.^50^ It is important to consider how to implement effective pain management in the ICU to reduce acute pain and reduce the risk of chronic pain in patients with COVID-19.^31,50^ Clinical practice guidelines for the prevention and management of pain, agitation/sedation, delirium, immobility, and sleep disruption in adult patients in the ICU have been developed and adopted in many ICUs.^14^ This strategy can obviously strengthen pain management of patients through multidisciplinary management of symptoms, mobility, and communication. Although there is not yet substantial evidence to support the statement that pre-existing chronic pain may worsen the pain during and after COVID-19, many severe chronic diseases such as coronary artery disease, diabetes, cancer, and chronic obstructive pulmonary disease are often accompanied by pain and also heighten the risk of death during COVID-19. Patients with chronic pain already experiencing depression, inattention, fatigue, and insomnia may find COVID-19 symptoms especially disturbing.

### 1.7. Presence of pain may affect the recovery from COVID-19

To concentrate available resources during the initial wave of COVID-19, most medical institutions had to close nonintensive care services including pain management departments.^55^ Besides the increased burden on the medical staff, patients with severe chronic pain, the disabled, and the elderly had reduced access to psychological and interdisciplinary treatments.^30^ A lack of face-to-face counseling services complicates individual assessment of analgesic use risk. When pain cannot be effectively assessed and treated, quality of life is reduced and treatment of underlying diseases may be impaired.^47,64^ The presence of chronic pain can activate the hypothalamus–pituitary–adrenal axis and alter the function of the immune and endocrine systems.^26,28,46,49,68^ At the Renmin Hospital of Wuhan University, a total of 663 patients with COVID-19 admitted between January 2020 and February 2020 were enrolled in a retrospective cohort study. Muscle pain was significantly associated with severe COVID-19 (*P* = 0.028) and to the patients' hospital course (*P* < 0.001). Muscle pain, expectoration, and decreased albumin were believed to be independent risk factors influencing patients' improvement during follow-up.^66^ Furthermore, groups such as the poor, the widowed elderly, and the homeless are more likely to suffer from chronic pain and comorbidities,^42^ have less access to health care, and are a high-risk group for severe COVID-19.^6^

Online pain management programs where patients are in contact with health care providers remotely are promising.^62^ Online resources can disseminate pain education and can develop online training programs on pain self-management for health care professionals, patients with pain, and relevant personnel. Although much of the existing research is optimistic about eHealth Care, studies may not adequately assess groups without access or ability to use these services, including children, the elderly, or those with intellectual disabilities.^16^

### 1.8. China's approaches to pain control during and after COVID-19

China's response to COVID-19 has been rigorous. After the outbreak, the Central Government's Leading Group for Epidemic Work swiftly formulated a response plan which put people's lives as the top priority. With the precise decision of the Chinese government and the joint efforts of volunteers from all over the country, the further expansion of the epidemic was finally controlled, which laid a solid foundation for the Chinese control of the epidemic throughout the country. Many of China's practices are worthy of discussion and reference.

### 1.9. Popularization of COVID-19 knowledge

Popularizing science and improving citizens' scientific quality are long-term concern of the Chinese government. This ensures that the public can properly understand and scientifically protect against the COVID-19 epidemic. In the very early days of the epidemic, China published the basic knowledge of novel coronavirus, the key points of self-protection, and the relevant national epidemic prevention and control policies to the public on the official government media, such as the Xinhua News Agency and China Central Television. At the same time, authoritative medical organizations, such as the Chinese Medical Association, China Preventive Medicine Association, and Chinese Association for the Study of Pain, educated the public through their official information release platforms. Through their professional guidance, self-health management of patients during the epidemic period was strengthened. For example, the Chinese Association for Science and Technology issued an article on home care and rehabilitation guidance for patients with shoulder joint pain during the epidemic.^13^ It is worth noting that new media platforms, which are more easily accepted by the people, have played a huge role in the prevention and control of the epidemic. Only when most people are familiar with the correct knowledge and prevention measures of COVID-19 can epidemic prevention work be performed more effectively.

### 1.10. Setting up professional diagnosis and treatment department

After the SARS epidemic and experience in 2003, China set up professional fever clinics in general hospitals grade II and above (grade III is the highest grade). All patients with fever, cough, and pain were to be admitted to the fever clinic for diagnosis and treatment. During the period of regular epidemic prevention and control, the registration, imaging examination, nucleic acid testing, isolation, reporting, treatment, and referral of patients with fever and pain can be conducted in this department. The fever clinics performed strict terminal disinfection operations. On December 3, 2020, the State Council's Joint Prevention and Control Mechanism Press Conference announced that more than 7,000 fever clinics had been completed nationwide.^45^

### 1.11. Standardize the use of analgesic and febrifuge drugs

Fever and pain are important early diagnostic indications for most patients with COVID-19. The China National Drug Administration issued an administrative order to further regulate the use of analgesic and antipyretic drugs, even those not typically thought of as drugs of abuse.^17^ Meanwhile, the National Health Committee issued the “Expert Consensus on the Management and Guidance of the Rational Use of Home Medications for Common People During the Epidemic Period”^18^ and provided pain management and pain medication training to all medical staffs. The purpose of these measures is to guide both medical staffs and patients on the correct use of medicines and correct reporting of early symptoms.

### 1.12. Early detection of close contacts

Finding close contacts of confirmed patients can help detect potentially infected persons by identifying trajectories and early symptoms, clearly describe the entire transmission chain and transmission network, and make epidemic control more effective.^25^ In January 2021, a few local cases were reported in Beijing, Shanghai, Hebei, and other places of China. The local governments immediately organized large-scale epidemiological follow-up. Most subsequent confirmed cases were close contacts identified by epidemiological means. The scattered outbreaks were controlled in only 1 month. Fever, pain, and other symptoms of patients with COVID-19 after infection clearing and discharge should also be included in the epidemiological follow-up.

### 1.13. Improve the public treatment system of COVID-19

In developing countries, even in many developed countries, the cost of treatment is a heavy burden for most patients with COVID-19. This may lead some patients to report their symptoms untruthfully. The Chinese government has designated special medical hospitals in each city to treat patients with COVID-19. In these institutions, free treatment including pain management for people infected with COVID-19 is provided. All the treatment costs are covered by the national public health insurance fund. This measure has helped implement the “early detection, early isolation, early treatment” guideline and fully guarantees the right of every citizen to have fair access to medical services. According to statistics from the China Medical Insurance Administration, in the first quarter of 2020 alone, China's public medical insurance invested more than 19.3 billion yuan (approx. 3.1 billion US dollars) for the treatment of patients with COVID-19.^52^ In addition, to encourage the public to receive the COVID-19 vaccine and ensure that low-income groups can also get the vaccine fairly, China has announced free vaccination for all citizens, the cost of which will be covered by the national public medical insurance fund.

China has adopted multiple channels to ensure the pain control needs of patients without COVID-19 are addressed. The National Ministry of Civil Affairs continues to purchase social medical services for poor people and actively encourages relevant companies to provide pain management services. For example, the Nationwide Hospice Service Program supported by Li Ka-shing Foundation provided free pain management and other services to approximately 15,000 patients with cancer pain during the COVID-19 epidemic.^12,35^

### 1.14. Adjust the measures of pain management

As COVID-19 has changed the original medical treatment model, pain management measures have been adjusted to minimize the impact of COVID-19 on patients with pain and the probability infection spread by patients with pain receiving unsupervised medical treatment. Based on the experience of effective prevention and treatment of SARS and MERS, 8 updated versions of Diagnosis and Treatment Protocol for Novel Coronavirus Pneumonia have been issued by the National Health Commission of China.^44^

The experts in the Department of Pain Medicine in Wuhan and Shenzhen have implemented a series of measures to take better care of the most vulnerable patients.^55^ After classification of diagnosis and treatment planning, outpatients with moderate and below pain received prescription drugs and received telemedicine support at home. Outpatients with severe pain were sent to clinic for rapid treatment. Any outpatients with confirmed or suspected COVID-19 infection will be sent to the quarantine area for further observation and treatment. For in-patients with severe pain, the necessary pain treatment was ensured as much as possible on the basis of minimizing contact.

Telemedicine is a convenient and safe way of providing pain management service during and after COVID-19, including instructions for taking medicine, relief of psychological symptoms, and at-home exercises.^55^ According to reports, the medical staff in Wuhan mobile cabin hospitals organized patients to conduct collective social activities, lead patients to practice square dances and increased theatrical performances.^61^

After discharge from hospital, patients should be followed up to see if sequelae are present.^67^ Recently, a book cowritten by Chinese doctors and scientific researchers in the field of COVID-19 prevention and treatment named *COVID-19: The Essentials of Prevention and Treatment* was released worldwide. This book recorded China's experience in prevention, control, and treatment for global reference.^51^

### 1.15. Promote traditional Chinese medicine treatment

Another highlight of COVID-19 treatment in China is the use of traditional Chinese medicine (TCM). Traditional Chinese medicine plays an important role in the treatment of patients with COVID-19 in China. China's Diagnosis and Treatment Protocol for Novel Coronavirus Pneumonia has included guidance on using TCM to treat patients with COVID-19 at all stages. The essence of Chinese medicine is applying medicine according to symptom-based diagnosis.^9^ The main symptoms of the patients with COVID-19 are fever, fatigue, pain, cough, myalgia/arthralgia, gastrointestinal symptoms, and “thick fur on tongue” which can be classified into dampness subtype on the basis of TCM theory.^10,11,65^ In TCM, the core pathogenesis of COVID-19 is the discomfort of the lungs and spleen caused by damp cold distemper. Early corresponding treatment should aim at eliminating moisture, thereby releasing lungs and eliminating pathogenic factors, so as to shorten the duration of fever, relieve symptoms including pain, and reduce mortality.^40^ There are many prescriptions and proprietary Chinese medicines which have been used in relieving COVID-19 symptoms in China. Most Chinese medicine guidelines define COVID-19 as an endemic, toxic, humid, or warm infectious disease.^48^ Licorice is the most frequently used Chinese medicine. Ephedra, bitter almond, astragalus, scutellaria, patchouli, and honeysuckle are also medicines that are frequently used. In Guangdong, a case study of Tou-jie-qu-wen granules was conducted and promoted to 30 designated hospitals as the standard treatment for patients with COVID-19.^22^ Pharmacological analysis showed that most of the active components of these herbs have antiviral, immunomodulatory, anti-inflammatory, and analgesic effects.^23^ In the process of treating patients with COVID-19, the use rate of traditional Chinese medicine in patients accounted for 90.6%, and the total effective rate was reported as over 90%.^52^

In addition, physicians use traditional nondrug methods such as acupuncture and massage as adjuvant therapy for patients.^67^ According to reports, stimulating the acupoint such as Tianzhu (BL10), BL12, and GV14 can eliminate nasal discharge, pain of the shoulders, and the back, while Neiguan (PC6) and Lieque (LU7), or Juque (CV14), Qımén (LR14), and Zhaohai (KI6) can relieve chest pain or/and shortness of breath.^37^ The traditional exercises named Taiji and Ba-Duan-Jin et al. are also used in rehabilitation training.^38^ The combination of TCM and modern medicine is used to treat patients with COVID-19 in China, giving full play to the respective possible strengths of TCM and modern medicine therapy.

## Disclosures

The authors have no conflicts of interest to declare.
